# Preferences for benefit packages for community-based health insurance: an exploratory study in Nigeria

**DOI:** 10.1186/1472-6963-10-162

**Published:** 2010-06-12

**Authors:** Obinna Onwujekwe, Chima Onoka, Nkoli Uguru, Tasie Nnenna, Benjamin Uzochukwu, Soludo Eze, Joses Kirigia, Amos Petu

**Affiliations:** 1 Department of Health Administration and Management, College of Medicine, University of Nigeria, Enugu-Campus, PMB 01129, Enugu, Nigeria; 2 Health Policy Research Group, Department of Pharmacology and Therapeutics, College of Medicine, University of Nigeria, Enugu-Campus, Enugu; 3 Department of Community Medicine, College of Medicine, University of Nigeria, Enugu-Campus, Enugu; 4 Department of Preventive Dentistry, College of Medicine, University of Nigeria, Enugu-Campus, Enugu; 5 World Health Organization, African Regional Office, Brazzaville; 6 World Health Organization, Nigeria Country Office, Abuja, Nigeria

## Abstract

**Background:**

It is important that community-based health insurance (CBHI) schemes are designed in such a way as to ensure the relevance of the benefit packages to potential clients. Hence, this paper provides an understanding of the preferred benefit packages by different economic status groups as well as urban and rural dwellers for CBHI in Southeast Nigeria.

**Methods:**

The study took place in rural, urban and semi-urban communities of south-east Nigeria. A questionnaire was used to collect information from 3070 randomly picked household heads. Focus group discussions were used to collect qualitative data. Data was examined for links between preferences for benefit packages with SES and geographic residence of the respondents.

**Results:**

Respondents in the rural areas and in the lower SES preferred a comprehensive benefit package which includes all inpatient, outpatient and emergencies services, while those in urban areas as well as those in the higher SES group showed a preference for benefit packages which will cover only basic disease control interventions.

**Conclusion:**

Equity concerns in preferences for services to be offered by the CBHI scheme should be addressed for CBHI to succeed in different contexts.

## Background

Many countries have implemented cost recovery systems, such as user fees, as a supplementary financing approach for health care services and this has raised concerns over equity and access to health care for the poor, and the search for "complementary financing solutions continues" [1]. The Commission on Macroeconomics and Health however recommends that out-of-pocket expenditures by poor communities should be channeled into community financing schemes to help cover the costs of community-based health delivery [2].

CBHI schemes have been around for a long time and in some cases have evolved out of traditional risk pooling mechanisms (such as the *tontine *in West Africa). A 1997 review identified 81 documented (CBHF) community based health-financing schemes throughout the world, with the majority in sub-Saharan Africa and Asia [3]. The number of CBHI initiatives is growing rapidly in sub-Saharan Africa. In West Africa, for example, the number of CBHI programs grew from 199 in 2000 to 585 that covered 1.5 million people in 2003 [4].

Previous studies have identified four main types of community based financing schemes [5]. These include: (i) community managed user fees where user fees relying on out-of-pocket health care payments are collected at the point of health care utilization; (ii) Community prepayment schemes where the community collects payments (in cash or kind) in advance, manages the funds collected, and pays the health care providers on behalf of its subscribers; (iii) Community provider-based health insurance where a provider serving a particular community collects the prepayments himself or herself from the subscribers and provides the needed health care to the subscribers; and (iv) Linked community health revolving fund - in this case the community acts as the agent of the government or social health insurance in reaching out to rural and excluded communities through contracts or some form of agreement [5]. Based on the Nigerian national health insurance scheme (NHIS) guidelines, and some other studies, the community pre-payment not-for- profit scheme has been proposed to be most relevant in Nigeria and in other "low income countries" [6,7] because it enables the members to decide on how and when to pay their premiums so that the scheme is responsive to their needs.

The use of community prepayment schemes has drawn a lot of interest in international health policy debates [8]. The fact that in many schemes communities participate in the process of defining the benefit package to be covered in advance (what to buy, in what form, and what to exclude) is a strength of CBHI [3]. Despite the appealing attributes of CBHI schemes, several operational difficulties have limited the success of several schemes [9]. Many of the shortcomings of CBHI relate to problems with scheme design, weak management and a lack of institutional capacity [3,10,11]. In Nigeria, such challenges could include scarcity of resources, limited experience with insurance mechanisms to pool and manage risk, and inefficient revenue collection, pooling and resource allocation and purchasing [12].

Poor access to health services and the predominant use of out of pocket payments and user fees as major forms of health financing has further worsened the health status of Nigerians making it difficult for the poor to get good healthcare and further impoverishing the people. This necessitated the creation of the Nigerian Health Insurance Scheme which is currently being implemented and was established to improve the health of Nigerians at an affordable cost. The benefit packages offered include basic outpatient and inpatient care, maternal and child health services etc though this only covers the formal sector. However the NHIS incorporates a community based financing scheme in order to take care of the informal sector [7].

To design a benefit package which is affordable, equitable and sustainable that will satisfy a varied number of persons has proven to be challenging because most CBHI schemes offer across board one benefit package [13], which mostly comprises curative services, generic drugs and uncomplicated deliveries [14]. This is because most insurers are not willing to take costly risks for small schemes largely because it endangers solvency when the number of claims rise [15]. And moreover to increase the benefit package will lead to an increase in the premium being paid.

Some studies in rural India have drawn attention to the fact that most schemes cover only primary care services and some form of hospital coverage [16,17] but when the participants were asked to choose, they preferred benefit packages which covered high aggregate costs like outpatient and inpatient admissions, drugs and laboratory tests regardless of the fact that they may not need those services frequently but felt it was important to them. [18]. This was observed in a scheme where referral services were offered and most of the participants chose cesarean section as one of the benefit packages [19].

It is important to carry along or seek the opinion of the participants in terms of benefit packages, premiums and also in defining conditions of the scheme, as previous studies have highlighted, the constant disagreement about what the poor want most - whether protection for emergencies, primary health care, drugs, surgeries, hospitalization or even transport cost [20]. If preferences are not determined, making benefit packages all inclusive (for example including HIV and Tuberculosis care) could result in adverse selection [20].

When CBHI schemes are developed, it is important that they are designed and implemented in such a way as to ensure the relevance of the benefit packages to beneficiary communities [5,21]. A review of 82 non-profit health insurance schemes for people outside formal sector employment in developing countries observed that very few of these schemes covered large populations or even covered high proportions of the eligible population [3]. For example, in a subset of 44 schemes the median percentage of the eligible population covered was 24.9%; 13 schemes had a coverage rate below 15%; and 12 schemes had a coverage rate above 50% [3].

The scope of benefit package offered by a CBHI affects the community response to its introduction, acceptability, enrollment and overall sustainability [21]. Pooling of risks within a community will vary along a spectrum covering two dimensions [22]. At one extreme in the spectrum of alternatives, a scheme could cover only very expensive medical care events that occur rarely (e.g., non-elective inpatient surgery) and at the other extreme, a CBHI scheme could cover only inexpensive medical care events that occur frequently (e.g., routine ambulatory consultations) [4,22,23]. Some CBHI schemes cover some combination of these extreme forms of risk pooling [21,22].

In order to enhance the potential of planned programmes to succeed, it is good practice to carry out studies to examine the feasibility of implementing such programmes. Using hypothetical scenarios, such studies can provide relevant information that will strengthen the programme design and implementation strategies. This paper examines the services that households being offered a hypothetical CBHI scheme would want to be included in the benefit package.

## Methods

### Study Area

The study took place in Enugu and Anambra states, southeast Nigeria. Enugu State has a population of 3,257,298 within a total area of 7,618 sq. km [24]. Enugu state is a well developed coal mining, commercial, financial and industrial centre, with a booming economy and vast investment opportunities [25]. Anambra state with its capital at Awka has a total population of 4,182,032 in a land area of 4,416 sq. km, giving an average density of 633 persons per sq. km. The people of the state are resourceful, hardworking, sociable, accommodating and are reputed for their business acumen [26].

### Study context and description of hypothetical CBHI scheme

The CBHI scheme that was explained to the respondents was the community prepayment type where a community-appointed committee collects payments prospectively from clients, manages the funds and purchases health care for clients from designated providers in the community. The choice of this form of CBHI was based on the fact that many communities in the study area usually undertake community development activities through management committees selected and empowered by community members, as was the case in the implementation of a district health system in Enugu state [27]. The committee would be regulated and managed by the government authorities. However, Anambra state is implementing a pilot CBHI scheme in 10 rural communities, which were excluded from this study. The benefit package used in the scheme would include preventive, curative and promotive services. Services would be delivered by health providers within the community registered with the scheme (including primary health centers, licensed patent medicine sellers, pharmacies and laboratories). An explanation of the benefit packages was given in detail to the respondents. By benefit package we mean a range of services an individual will be willing to pay for if he or she is enrolled in the health insurance scheme. Benefit package is considered comprehensive if it covers everything (including all inpatients and outpatient services and emergencies but the inpatient care will be limited to 45 days per year in a standard ward). The benefit package may be partial where it covers only a few diseases that are most common in the area including malaria, typhoid, diarrhea, TB and hypertension.

#### Study Design

This was a cross-sectional study using a pre-tested interviewer-administered questionnaire (Additional file 1) to collect information from randomly selected households. There were also focus group discussions (FGDs) with community members. The study was conducted in three communities in each state. These were an urban, a semi-urban and a rural community. The selection of the three different types of communities was done to present a broad picture of acceptability of CBHI in different settings. In Anambra state; Awka (urban), Amawbia (semi-urban) and Amansea (rural) were used as the study areas. In Enugu state; Uwani (urban), Iji-Nike (semi-urban) and Amokwe (rural) were the study communities.

### Focus group discussion (FGD)

In each study site, FGDs were stratified by sex (male and female). The combination yielded a total of 2 FGDs per site and 12 in total. There were about 9-10 members in each FGD and each lasted between45 and 60 minutes. The participants were purposively selected so that all sections of each community were represented. A question guide was used to elicit information on the theme of the study which was used to understand the factors that explain acceptability and preferences for the benefits offered by CBHI.

### Household Survey

The interviewers were trained over a period of three weeks so as to ensure their mastery of the questionnaire and on issues about health insurance. The questionnaire was administered to respondents from a total of 3070 households. A minimum sample of 500 households was drawn from each community using simple random sampling technique. Adequate sample size was determined, using a power of 95% confidence level and utilization rate of health facilities of 20%. In order to account for refusals, the sample size was increased to 520 for each study community giving a total of 3120 households.

The heads of households or the most senior member of the household from the selected households were interviewed. The meaning of health insurance, CBHI and the purpose of the study was explained to the respondents. All areas of concern or confusion about the study were then clarified by the interviewers, after which consent was obtained. Data was collected on the demographic and socioeconomic characteristics of the population. The ranking of preferences for benefit package was determined thereafter. However the brief explanation about health insurance and its attributes was provided to the respondents before determining their levels of preferences. They were asked to rank their preferences for the different health insurance benefit packages on a scale of 1 (least preferred) to 5 (most preferred). A monthly premium rate of 500 Naira was proposed to the respondents and they were now asked how much they would be willing to pay. Their WTP was elicited using the bidding game question format of the contingent valuation method. The questions were structured in English Language for the elicitation of information from the respondents and translated to the native Igbo language for actual questioning.

### Data Analysis

The data was merged to give data sets for urban, peri-urban and rural areas. Data was examined for links between key dependent variables with socio-economic status (SES) and geographic location of the respondents. Principal component analysis technique was employed to generate a socio-economic status index [28] which was used to categorize households into quartiles: Q1 (most poor); Q2 (very poor); Q3 (poor) and; Q4 (least poor) for examination of SES differences in the variables. This index was generated using information gathered from the participants on ownership of a radio, television, refrigerator, bicycle, motor car, and motorcycle together with the weekly per capita cost of food. Data analysis was done using STATA and SPSS software.

Comparison between the three geographic locations was used to examine for geographic differences. Content analysis was used to analyse the FGD data.

### Ethical Clearance

This research was approved by the ethical clearance committee of the College of Medicine University of Nigeria, Enugu Campus Enugu State Nigeria.

## Results

### Socio-demographic characteristics of the respondents

Table 1 shows that in the overall sample of 3070, most of the respondents from the six study communities were male heads of households (61.7%), were in their forties, main income earners (83%) and main decision makers about household expenditures (84.7%). The table also shows that the average number of household residents was five people in the six study areas.

**Table 1:** Socio-demographic characteristics of the respondents by communities

Variables	Awka n = 500	Amawbia n = 500	Amansea n = 500	Uwani n = 515	Iji-Nike n = 555	Amokwe n = 500
Male household head: n (%)	334 (66.8)	263 (52.6)	313 (62.6)	180 (35.0)	404 (72.9)	401 (80.2)
Whether respondent is main income earner: n (%)	460 (92.0)	386 (77.2)	463 (92.6)	233 (45.2)	526 (94.8)	480 (96.0)
Whether respondent is main decision-maker: n (%)	457 (91.4)	398 (79.6)	469 (93.8)	252 (48.9)	545 (98.2)	481 (96.2)
Sex (Male): n (%)	352 (70.4)	270 (54.0)	309 (61.8)	183 (35.5)	403 (72.6)	397 (79.4)
Mean number of household residents (SD)	4.93 (4.91)	5.09 (4.87)	4.93 (2.85)	5.49 (2.49)	5.48 (4.25)	5.42 (2.16)
Mean age( years) of respondent (SD)	44.76 (15.43)	47.26 (14.58)	43.71 (11.24)	41.65 (12.96)	41.85 (12.08)	49.16 (12.44)

### Health care benefits expected from CBHI (FGD)

The respondents mentioned a lot of benefits as follows: Proper treatment with less amount of money; would not need to have money at hand before going to the hospital; could easily take any member of my family to the hospital; will reduce patronage of informal providers; will ensure quick access to hospital if our children are sick; and would help the poor live longer.

According to some of the discussants: *What we are hoping to benefit from the project is that when you or your child falls sick and if you don't have money, it will serve as shock-absorber to the family (***FGD Female Per-urban community)**

*If the community can organize the CBHI in such a way that maternity issues will be given attention so that child birth will no longer be a matter of life and death because this is a problem (***FGD Male urban community***)*

### Preferences for benefit packages

Table 2 shows that the highest ranking was for a benefit package that will cover everything (i.e. disease control, outpatient and inpatient services, and emergencies), followed closely by a benefit package that will cover only out-patient services.

**Table 2:** Average ranking of preferences for different CBHI benefit packages in the three types of communities

Variables	Urban Mean (SD)	Peri-urban Mean (SD)	Rural Mean (SD)	X2 (p-value)
a.Covers everything	4.1 (1.2)	4.0 (1.2)	4.3 (1.2)	25.1 (p < .05)
b.Covers only basic disease control	3.0 (1.2)	2.6 (1.3)	2.7 (1.2)	76.3 (p < .05)
c.Covers only outpatient services	3.4 (1.2)	3.9 (1.2)	3.6 (1.0)	133.2 9p < .05)
d.Covers only inpatient services	1.8 (1.0)	2.1 (1.0)	1.9 (1.0)	60.4 (p < .05)
e.Covers only emergencies	2.6 (1.3)	2.4 (1.1)	2.6 (1.2)	14.3 (p < .05)

a*all inpatient and outpatient services and emergencies

b* prevention and treatment of common illnesses such as malaria, typhoid, diarrhea, etc

c* outpatient care including necessary consumables such as essential drugs and essential diagnostic tests, maternity care for up to four live births, preventive care such as immunization, health education, family planning antenatal and postnatal care, cosulation with specialist such as paediatricians, obstetricians, gynaecologists and general surgeons

d*Hospital care in a standard ward for stay limited to 45 days per year

e* emergency obstetric care , accidents and traumas, medical emergencies such as diabetic coma, stroke and heart attack

In the FGD, the consensus opinion of the participants was that CBHI should cover both out-patient visits and in-patient stays. The consensus opinion was also that the benefit package should include common communicable (TB, chicken pox, malaria, Typhoid fever, whooping cough, measles etc and non-communicable ( hypertension, diabetes, cancers, injuries, accidents, etc.) diseases.

Table 3 shows that the strength of preference for a comprehensive benefit package was highest in the rural areas. The urban dwellers showed higher preferences when compared to peri-urban and rural dwellers for a benefit package that will cover only basic disease control interventions (This will cover the prevention and treatment of common illness such as malaria, typhoid, diarrhea etc). The most-poor SES showed higher preferences for benefit package that will cover everything. Conversely, the least poor SES group had the least score for the comprehensive benefit package and one of the highest scores for a benefit package that will cover only basic disease control interventions.

**Table 3:** Average ranking of preferences for different CBHI benefit packages by socio-economic status (SES)

Variables	Q1 (most poor) Mean (SD)	Q2 (very poor) Mean (SD)	Q3 (poor) Mean (SD)	Q4 (least poor) Mean (SD)	X2 (p-value)
Covers everything	4.2 (1.2)	4.2 (1.2)	4.2 (1.2)	4.0 (1.2)	11.9 (p < .05)
Covers only basic disease control	2.6 (1.2)	2.7 (1.2)	2.8 (1.3)	2.8 (1.2)	16.7 (p < .05)
Covers only outpatient services	3.7 (1.1)	3.8 (1.1)	3.5 (1.2)	3.6 (1.3)	16.1 (p < .05)
Covers only inpatient services	1.9 (1.0)	1.9 (1.0)	2.0 (1.1)	2.0 (1.0)	4.8 (p > .05)
Covers only emergencies	2.6 (1.3)	2.4 (1.2)	2.5 (1.2)	2.6 (1.3)	9.5 (p < .05)

## Discussion

The finding that most people preferred a benefit package that covers everything including all in-patient and outpatient services is not surprising. Previous studies similarly found strong preferences for inclusion of high-cost health services such as surgical operations as well as low cost items such as essential drugs and consultation fees [5]. The preferred benefit package may not be actualized in the actual implementation because they will be based on the financial resources available for running the scheme which is dependent on the number of people enrolled and premiums paid by members of the scheme. However, health care benefit packages covering the major causes of ill-health in a target community should be encouraged, since they ensure that those in need derive optimal benefit from health services and receive value for the money spent on these services [22].

Next to covering everything option, most respondents preferred for the CBHI with benefit package to cover only outpatient services. This may be because outpatient services only may be seen as the common health care need of most households. This is strengthened by the finding that the package covering only the inpatient services had the least proportion of people who preferred it most, indicating that inpatient services may be the lowest item in the priority list if the establishment of a CBHI scheme is contemplated in areas where limited funds need to be used effectively. This must however be done with caution since results from studies in Asia indicate the best financial protection is provided by widespread risk pooling, minimal user fees and benefit packages that cover hospitalization [29].

Irrespective of the package covering various health services finally selected, the basic benefits of financial risk protection, quick access to care and benefits, and reduction in cost of treatment must be ensured in the scheme design once the target population accepts the idea of the CBHI scheme [30]. This will promote long term trust in the scheme, which is necessary for sustainability of such schemes. More importantly, the process of making decisions on the benefit package must involve the people as insurance schemes have been known to have had to repeatedly review the benefit package and premiums upon receiving opinions of their beneficiaries [2].

### Limitations

The different benefit packages had no price tag attached to each of them which might not give the respondents a clear picture of which package to choose. However all the respondents were informed of the different monthly premiums they might have to pay and were also made to understand that the benefit packages were tied to the premiums and the more comprehensive the package the higher the premium this information. This is a hypothetical willingness to pay study and may not reflect actual practice. A follow up study following introduction of the scheme in the study area will provide very useful information about revealed preferences. Again the paper was only interested in the benefit packages the potential beneficiaries would want included in the CBHI scheme when in operation so the analysis did not consider the wealth quartiles and their particular preferences in the three types of communities used in this study. Furthermore, the use of durable assets in generating the index for both rural and urban is a limitation; however, the study applied a common denominator in assessing wealth in southeast Nigeria (rural or urban) which is based on durable assets and household weekly food consuption as used in this study.

Future studies: Discreet choice experiment (DCE) with price tagged to different benefit packages; Differential WTP for different benefit packages.

## Competing interests

The authors declare that they have no competing interests.

## Authors' contributions

OO conceived the study. All the authors participated in data collection and analysis. OO and NU wrote the manuscript with input from all the authors. All authors read and approved the final manuscript

## Pre-publication history

The pre-publication history for this paper can be accessed here:

http://www.biomedcentral.com/1472-6963/10/162/prepub

## Supplementary Material

Additional file 1 **Household Questionnaire**. Contains a household questionnaire on feasibility of community based health insurance. It contains demographic information of the respondent, health seeking pattern of the household, payment and payment coping mechanisms. It also contains questions about acceptability and willingness to pay for CBHI scheme, perceptions about the scheme and preferences for different benefit packages. Questions on household consumption patterns and asset holdings were also included in the questionnaire. Click here for file
