# Supplementary material for: Preferences for benefit packages for community-based health insurance: an exploratory study in Nigeria
Source: BMC Health Serv Res. 2010 Jun 12;10:162. doi: 10.1186/1472-6963-10-162 (PMC2896948; doi:10.1186/1472-6963-10-162)
Supplement: Additional file 1 — Household Questionnaire. Contains a household questionnaire on feasibility of community based health insurance. It contains demographic information of the respondent, health seeking pattern of the household, payment and payment coping mechanisms. It also contains questions about acceptability and willingness to pay for CBHI scheme, perceptions about the scheme and preferences for different benefit packages. Questions on household consumption patterns and asset holdings were also included in the questionnaire. [file 1472-6963-10-162-S1.DOC]

## **Household Questionnaire: Feasibility of Community-Based Health Insurance**

## Dear respondent,

I am a trained local worker sent by the Enugu/Anambra State Ministry of Health and College of Medicine, University of Nigeria Enugu to elicit your opinion about the development of community-based health insurance program in your community. As you probably know, high cost of health care prevents many people from accessing much needed health care everyday. It is an issue that requires immediate strategic attention. Hence, we wish to interview you regarding healthcare seeking habits, as well as hear any suggestions you have for reducing the burden of healthcare cost for the people of your community. We would also like to understand your knowledge and willingness to participate in a community-based health insurance program. Be assured that your identity will be kept confidential during the research process. Your participation is voluntary and you do not have to answer any questions you do not want to, however it will help in the establishment of a health insurance scheme in your community, Anambra/Enugu State and Nigeria. If you have any questions you may ask them now or later.

***(Enumerator: Prior to beginning the interview, read the consent form on the last page to the respondent. Sign that they have been read the form, and give them a copy of the information sheet).***

***Instruction: Please fill the next section at the beginning of the interview.***

## **Pre-Interview Information**

### This part should be filled by the enumerator before the interview

Code number: _____________

What is the respondent's home address_____________________________________________________________

Name of the interviewer: ___________________________________________________________________________

What is the date of interview < / / >

What time did the interview start: ________________________

*Please, enter the appropriate number representing the answer given in the spaces provided. Please note that throughout the questionnaire, if YES write 1 in the box, if NO, write 0 in the box.* ***All boxes MUST be filled.***

## **SECTION 1: DEMOGRAPHIC INFORMATION**

***Enumerator read****:* This section is designed to help us learn about you and your household. Please take a few minutes to answer the following questions.

1. What is your name: ______________________________________________

2. What is your status in this household? [ ] 0 = female head of household; 1 = male head of household; 2 = wife; 3 = grandmother; 4 = representative of household

*[Enumerator: Only the head of the household should be interviewed or in his/her absence the spouse should be interviewed or another adult income earner].*

3. Are you the main income earner in your household? [ ] 1 = yes 0 = no

4. Do you consider yourself to be the main decision-maker in your household about what your household spends money on? [ ] 1 = yes 0 = No

5. How many people live in this household, including yourself? [ ]

6. How many adults (greater than or equal to 18 years) live here? [ ] No. of adults

7. How many younger people (less than 18 years) live here? [ ] No. of other people

8*. (Enumerator: Add the responses in questions 6-7 and see if they add up to question 3 response)* [ ] Total of questions 6-9

9. How old are you? [ ]

10. *Enumerator: Respondent’s Sex?* [ ] 1 = male 0 = female

11. Did you go to school? [ ] 1 = yes 0 = no (IF YES, GO TO QUESTION 12, IF NO, GO TO QUESTION 13)

12. What was your highest completed education level? 1 = yes 0 = no

12a. Primary [ ]

12b. Junior Secondary [ ]

12c. Senior Secondary [ ]

12d. Teachers Training College [ ]

12e. College of Education [ ]

12f. University or Polytechnic [ ]

12g. Others [ ] __________________________________________________

13. What was the total number of years that you spent schooling? [ ]

14. What occupation is your major source of income? 1 = yes 0 = no

14a. Farmer [ ]

14b. Unemployed [ ]

14c. Petty trading [ ]

14d. Government Worker [ ]

14e. Employed in private sector [ ]

14f. Big business [ ]

14g. Self-employed professional [ ]

14h. Others [ ] Please, specify: ________________________________________________

15. What occupation is the household’s major source of getting money? [ ] *[Enumerator: Note that it may or may not be respondent’s occupation]*

**SECTION 2A: HEALTH SEEKING AND COST OF ILLNESS FOR HEAD OF HOUSEHOLD**

*I want to talk with you about the most recent episode of illness or health condition (within the past month).*

16. What was the most recent type of sickness or poor health condition you had within the past month? 1 = yes 0 = no

16a. Malaria [ ]

16b. Typhoid [ ]

16c. Diarrhea [ ]

16d. Other [ ] Please specify: __________________________________________

17.Where did you first seek treatment? 1 = yes 0 = no *[Enumerator: Do not read list. Mark first response only.] (Write down the name and address of the provider(s) visited)*

17a. Traditional medicines: [ ] ____________________________________________________________

17b. Chemist (patent medicine dealer): [ ] ______________________________________

17c. Community health worker: [ ] _______________________________________________

17d. Health center: [ ] _________________________________________________________

17e. Public (general) hospital or clinic: [ ] _______________________________________________

17f. Private hospital or clinic: [ ] _______________________________________________________

17g. Other: [ ] please specify: ______________________________________________________________

18. What form of transportation did you use to reach the location where you obtained treatment? 1= yes 0 = no

18a. Personal vehicle: [ ]

18b. Bus (public transport) [ ]

18c. Taxi [ ]

18d. Okada [ ]

18e. Walked [ ]

18f. Others [ ] Please specify _______________________________

19. How long did it take to get to the location of treatment? 1= yes 0 = no

19a. Less than 15mins [ ]

19b. 15-30mins [ ]

19c. 30-1hour [ ]

19d. greater than 1 hour [ ]

19e. greater than 2 hours [ ]

20. Approximately how many minutes did it take you to get to where you received treatment [ ] mins

21. How much did you spend on transportation to receive this treatment (to and fro)? _________________ Naira

22. How much did it cost to receive this treatment (including cost of registration/card, cost of drugs, laboratory tests, x-rays, etc)? ___________ Naira

23. Total cost [*Enumerator add 21 +22]* _________________ Naira

24. Did you have to remain in the health facility visited for more than one day? [ ] 1 = yes (go to Q25) 0 = no (go to Q26)

25. *If yes to question 24*. How many days did you remain at the facility? ­ ________________

25. Did you recover after receiving this treatment? [ ] 1 = yes 0 = no *[Enumerator: If YES, go to Section 2B, if NO, go to question 26]*

**26. Where did you go to receive further treatment and what was the total cost you spent (transportation/drugs/others) until you recovered? *(Enumerator: Complete the table using all the information provided)***

|  | Place visited | Diagnosis | Total cost |
| --- | --- | --- | --- |
| 26a. 2^nd^ action |  |  |  |
| 26b. Other actions until recovery |  |  |  |
| 26c.**Total 24a+24b+22)** |  |  |  |

**SECTION 2B: PAYMENT AND PAYMENT COPING MECHANISMS FOR RESPONDENT**

27. How was the treatment cost paid for the first treatment? I will read out some payment options and please answer either yes or no to each option. 1 = yes 0 = no *[Enumerator: Multiple responses are allowed]*

27a. Paid (cash and carry) but was reimbursed by employer [ ]

27b. Out-of pocket cash and carry [ ]

27c. Health Insurance [ ] Please specify type ______________________________________________

27d. Installment [ ]

27e. In-kind [ ] Please specify ______________________________________

27f. Others (please specify) [ ] _______________________________________

28. How did you cope with the payment for the treatment? I will read out some options and please answer either yes or no to each option. *(Enumerator: Multiple responses are allowed)*Coding of payment coping mechanisms 1 = yes 0 = No

28a. Own money [ ]

28b. Borrowed money/took a loan [ ] Please specify source of the loan ______________________________

28c. Sold household movable assets [ ]

28d. Sold family land [ ]

28e. Payment was subsidised [ ]

28f. Payment was deferred [ ]

28f. Community solidarity/someone else paid [ ]

28h. Was exempted from payment [ ]

28i. Others (specify) ___________________________________

29. How easy was it for you to use the following payment strategies? *[Enumerator: Multiple answers are allowed]* 1 = easy 0 = not easy 2 = Not applicable

29a. Paid (cash and carry) but was reimbursed by employer [ ]

29b. Out-of pocket Cash and carry [ ]

29c. Health Insurance [ ] Please specify ______________________________________________

29d. Installment [ ]

29e. In-kind [ ] Please specify ______________________________________

29f. Others (please specify) [ ] _______________________________________

**Enumerator for those that used any form of health insurance, ask question 30**

30. Why did you or your household take out a health insurance policy? I will read out some options and please answer either yes or no to each option. *(Enumerator: Multiple responses are allowed)* Coding of reasons focusing health insurance 1 = yes 0 = No

30a. Financial protection against the cost of illness [ ]

30b. Provide access for household members to affordable healthcare [ ]

30c. Provide good quality treatment for reduction of income loss due to ill health [ ]

30d. Others [ ] Please specify ____________________________________________

**SECTION 2C: HEALTH SEEKING (OTHER HOUSEHOULD MEMBERS)**

Now I'd like to ask you about other household members besides yourself and their experience with paying for healthcare.

31. In total, how many *other household members were* sick over the past 1 month? ______ No. of household members (*Enumerator: If nobody was ill or had a health condition, go to Section 3).*

32. What was the most recent type of sickness or poor health condition of the last household member that was ill?

1 = yes 0 = no

32a. Malaria [ ]

32b. Typhoid [ ]

32c. Diarrhea [ ]

32d. Other [ ] Please specify: __________________________________________

33.Where did you first seek treatment? 1 = yes 0 = no *[Enumerator: Do not read list. Mark first response only.] (Write down the name and address of the provider(s) visited)*

33a. Traditional medicines: [ ] ____________________________________________________________

33b. Chemist (patent medicine dealer): [ ] ______________________________________

33c. Community health worker: [ ] _______________________________________________

33d. Health center: [ ] _________________________________________________________

33e. Public (general) hospital or clinic: [ ] _______________________________________________

33f. Private hospital or clinic: [ ] _______________________________________________________

33g. Other: [ ] please specify: ______________________________________________________________

34. Did the person have to remain in the health facility visited for more than one day? 1 =yes 0 = no ____________

35. What form of transportation did you use to obtain treatment the member of your household? 1= yes 0 = no

35a. Personal vehicle: [ ]

35b. Public Transport (bus) [ ]

35c. Taxi [ ]

35d. Okada [ ]

35e. Walked [ ]

36. How long did it take to get to the location of treatment? 1= yes 0 = no

36a. Less than 15mins [ ]

36b. 15-30mins [ ]

36c. 30-45mins [ ]

36d. 45-1hour [ ]

36e. greater than 1 hour [ ]

36f. greater than 2 hours [ ]

37. How much did you spend on transportation to receive this treatment? _________________ Naira

38. How much did it cost to receive this treatment (including cost of registration/card, cost of drugs, laboratory tests, x-rays, etc)? ___________ Naira

39. Total cost [*Enumerator add 37 +38]* _________________ Naira

40. Did you recover after receiving this treatment? [ ] 1 = yes 0 = no *[Enumerator: If YES, go to Section 2D, if NO, go to question 41]*

**41. Where did you go to receive further treatment and what was the total cost you spent (transportation/drugs/others) until you recovered? *(Enumerator: Complete the table using all the information provided)***

|  | Place visited | Diagnosis | Total cost |
| --- | --- | --- | --- |
| 41a. 2^nd^ action |  |  |  |
| 41b. Other actions until recovery |  |  |  |
| 41c.**Total (41a+41b+39)** |  |  |  |

*42. Table summarizing households’ expenditure to treat other household members’ illnesses in last month.* (Enumerator: Complete the table using all the information provided above)

|  | Where visited | Diagnosis | Total cost |
| --- | --- | --- | --- |
| 42a. 1^st^ action |  |  |  |
| 42b. 2^nd^ action |  |  |  |
| 42c. Other actions until recovery |  |  |  |
| 42d. Total |  |  |  |

**SECTION 2D: PAYMENT AND PAYMENT COPING MECHANISMS FOR OTHER HOUSEHOLD MEMBERS**

43. How was the treatment cost paid? I will read out some options and please answer either yes or no to each option. = yes 0 = no *[Enumerator: Multiple responses are allowed]*

43a. Paid (cash and carry) but was reimbursed by employer [ ]

43b. Out-of-pocket Cash and carry [ ]

43c. Health Insurance [ ] please specify _______________________________________________

43e. Installment [ ]

43f. In-kind [ ] Please specify ______________________________________

43h. Others (please specify) [ ] _______________________________________

44. How did you cope with the payment? I will read out some payment options and please answer either yes or no to each option. *(Enumerator: Multiple responses are allowed)* Coding of payment coping mechanisms [ 1 = yes 0 = No]

44a. Own money [ ]

44b. Borrowed money [ ] Please specify source of the loan _____________________________________

44c. Sold household movable assets [ ]

44d. Sold family land [ ]

44e. Payment was subsidised [ ]

44f. Payment was deferred [ ]

44g. Community solidarity [ ]

44h. Someone else paid [ ]

44i. Was exempted from payment [ ]

44j. Others (specify) ___________________________________

**Enumerator for those that used any form of health insurance, ask question 45**

45. Why did you or your household take out a health insurance policy? I will read out some options and please answer either yes or no to each option. *(Enumerator: Multiple responses are allowed)* Coding of reasons focusing health insurance 1 = yes 0 = No

45a. Financial protection against the cost of illness [ ]

45b. Provide access for household members to affordable healthcare [ ]

45c. Provide good quality treatment for reduction of income loss due to ill health [ ]

45d. Others [ ] Please specify ____________________________________________

**SECTION 3: ACCEPTABILITY OF CBHI INSURANCE SCHEME (WILLINGNESS TO ENROLL IN CBHI)**

***Enumerator read***: Now I would like to ask you a few questions regarding your preferences and acceptability of health insurance. Health insurance is method used to pay for health care that helps decrease the uncertainty that may accompany inability to pay for healthcare at the time it is needed. I will briefly introduce health insurance before asking you a few questions.

**Introduction to health insurance:** Health insurance is a program that pools the risk of several people in an effort to decrease the amount that is paid by an individual at the time health care services are needed. Ill-health occurrence is largely unpredictable for individuals, as such, the need for health-care is often highly unpredictable and very costly for most individuals, however, it is predictable for large groups. Health insurance provides an opportunity to spread the financial burden of payment over several people thus making health care more affordable for individuals.

***[Enumerator: Ask whether respondent has any question]***

Health insurance can be either compulsory or voluntary. Compulsory health insurance is normally found within a obligatory public scheme. Voluntary health insurance can be offered by a private entity, public or quasi-public body. Health insurance can also be offered at a community-level and is known as *community-based health insurance. It is*  usually voluntary, but could be compulsory if the community so decides. In *community-based health insurance scheme,* the people of the community pay a predetermined amount and depending on the benefits packages available, will have access to various health providers and services.

Different benefit packages are offered by different insurance programs depending on the amount of contributions (premium) paid by the consumers. Benefits packages can vary widely from coverage of every healthcare need to covering only essential healthcare needs. The greater the number of services covered in the package, the greater the contribution required of the consumers.

The major parties in health insurance scheme include the consumers, the providers, and the third party financers (insurers). Their relationship with one another that establishes the framework for the insurance plan. Theses interactions can be determined by the community or any other entity designated to determine benefits. The providers include: medical doctors, pharmacists, nurses, and other allied health practitioners.

***[Enumerator: Ask whether respondent has understood the subject matter]***

**SECTION 3A: PERCEPTIONS ABOUT THE USE OF COMMUNITY-BASED HEALTH INSURANCE TO IMPROVE PAYMENT FOR HEALTHCARE**

***EXPLANATION OF WHAT CBHI IS:***

Community- based health Insurance (CBHI) is a non-profit type of health insurance for the informal sector, formed on the basis of an ethic of mutual aid and the collective pooling of health risks, in which members pay small premiums on a regular basis to offset the risk of needing to pay large health care fees upon falling sick. However, unlike many insurance schemes, CBHI schemes are typically based on the concepts of mutual aid and social solidarity and are useful for people in the informal and rural sectors and even those formally employed who are unable to get adequate public, private, or employer-sponsored health insurance.

***[Enumerator: Ask whether respondent has any question]***

Membership in a scheme is voluntary and evidence suggests that that it is primarily the rural middle class that joins such schemes. CBHI provides some financial protection by reducing out-of-pocket spending (OOPS), improves cost-recovery and could lead to improvement in quality of services. Hence, CBHI schemes are seen as a promising tool for the improvement of the health system of communities in low income countries. The prevailing health financing mechanism in Nigeria, which is OOPS have been found to impose heavy burden on households and it severely impedes access to health care utilization by particularly by those who stand in greatest need of care – the poor. CBHI offers opportunity for coverage of many people outside the formal sector, and if well designed, may be inclusive of the poor.

***[Enumerator: Ask whether respondent has understood the subject matter]***

46. From either your experience or understanding, how would you rate the potential financial protection by community-based health insurance against the cost of illness? [ ] 0 = none 1 = low 2 = medium 3 = high

47. What do you think is the potential level of access by households to affordable healthcare due to community-based health insurance? [ ] 0 = none 1 = low 2 = medium 3 = high

48. How would you rate the potential of community-based health insurance to improve household health consumption patterns by ensuring that healthcare costs are reduced? [ ] 0 = none 1 = low 2 = medium 3 = high

49. How would you rate the potential of CBHI to improve the quality of services provided by healthcare givers? [ ]

[ ] 0 = none 1 = low 2 = medium 3 = high

50. How would rate the potential of CBHI to ensure constant availability of drugs at health facilities in your community?

[ ] 0 = none 1 = low 2 = medium 3 = high

**SECTION 3B: ACCEPTIBILITY OF COMMUNITY-BASED HEALTH INSURANCE**

51. Is community-based health insurance acceptable to you as a strategy for paying for health care in this area? [ ]

1 = yes (go to Q52) 0 = no (go to Q53).

52. If Q50 is yes, please score your level of acceptability of CBHI from 1 to 10 [ ] where 1 is least preferred and 10 is most preferred.

53. If CBHI is not acceptable, why? _________________________________________________________________

**SECTION 3C: PREFERENCES FOR DIFFERENT BEENFIT PACKAGES**

54. Please, rank how you prefer the different health insurance benefit packages. Ranking is from 1 (least preferred) to 5 (most preferred). SO YOU CAN RANK 1,2,3,4,5. (*Enumerator read out options).*

54a. Covers everything (including all inpatient and outpatient services and emergencies) [ ]

**What is covered:** This will cover all forms of outpatient and inpatient care, but the inpatient care will be limited to a cumulative stay of 45days per year in a standard ward.

54b. Covers only basic disease control services [ ]

**What is covered:** This will cover the prevention and treatment of common illness such as malaria, typhoid, diarrhea etc.

54c. Covers only outpatient services [ ]

**What is covered:** (1) out-patient care including necessary consumables; (2) Essential drugs and essential diagnostic tests; (3) Maternity care for up to four live births; (4) Preventive care such as immunization, health education, family planning, antenatal and postnatal care; (5) Consultation with specialists such as physicians, pediatricians, obstetricians, gynaecologists, general surgeons.

54d. Covers only inpatient services [ ]

**What is covered:** Hospital care in a standard ward for stay limited to cumulative 45 days per year

54e. Covers only emergencies [ ]

**What is covered:** It will cover emergency obstetric care, accidents and traumas, medical emergencies such as diabetic coma, stroke and heart attack etc

**SECTION 4: WILLINGNESS TO PAY (WTP) FOR COMMUNITY-BASED HEALTH INSURANCE**

***Enumerator:*** Now, I would like to ask you about your level of WTP for a proposed community-based health insurance scheme for yourself and other family members.

**Scenario for eliciting WTP for community-based health insurance**

The benefit package in the proposed insurance will consist of a basic health package, which includes all primary healthcare (i.e. routine health maintenance, treatment of common illness such as malaria, typhoid, diarrhea), some aspects of secondary care (i.e. surgery, and hospital admissions, emergencies and other acute lifesaving investigations and operations). Healthcare would be delivered by healthcare providers (hospitals, pharmacists, laboratories, etc.) in your local area which is registered with the community-based health insurance management group.

In more detail, the benefit package in the proposed insurance plan covers selected preventive, curative and promotive health services and they include: (1) out-patient care including necessary consumables; (2) Essential drugs and essential diagnostic tests; (3) Maternity care for up to four live births; (4) Preventive care such as immunization, health education, family planning, antenatal and postnatal care; (5) Consultation with specialists such as physicians, pediatricians, obstetricians, gynaecologists, general surgeons etc.; (6) Hospital care in a standard ward for stay limited to cumulative 45 days per year (7) Eye examination and care excluding provision of spectacles and contact lenses; and (7) Preventive dental care and pain relief.

***[Enumerator: Ask whether respondent has any question]***

The CBHI will be managed by a committee or a management group that your community will establish, supported by the state government and development partners. The CBHI management group will be regulated and guaranteed by the state government to ensure that they maintain high quality corrupt- free operations and ensure that people’s contributions are safe. Your community’s management team will be solely responsible for the allocation of payments and other various administrative aspects of running the program.

You will be required to pay a premium in order to join the scheme. The premium can be paid monthly or once a year, however, no credit is allowed. Flat-rate premiums would be used and the premium should be paid directly to CBHI management team in your community. All premiums will be overseen by the CBHI management group. After payment of the premium, the enrollees will receive services free of charge at the point of consumption because they have already paid their premium.

***[Enumerator: Ask whether respondent has any question]***

Each registered person will be issued with an identity cared renewable yearly after payment of premium. The services will be accessed from primary healthcare centres, general hospitals, selected private hospitals and from mission hospitals that the management committee will register. The consumers are restricted to the providers which the CBHI management group will select.

Please, be aware that you will be free to continue receiving health care as a you presently do without necessarily taking a health insurance policy.

55. Will you be willing to enroll in the community-based insurance scheme? [ ] 1= Yes 0= No

55a. If no why _____________________________________________________________

56. Will you be willing to enroll other household members in the community-based health insurance scheme? [ ]

1 = Yes 0 = No

56a. If no why _____________________________________________________________

**We would now like to know the maximum amount of money that you will be willing to pay for yourself and for other members of your household (each to have the community-based health insurance coverage).**

**57. WTP FOR SELF**

1. The price of a monthly insurance premium (contribution) is 500 Naira; are you willing to pay? [ ] 1 = Yes (Q7) 0 = No (Q2) Do not know (Q2)

2. What is the maximum amount you are willing to pay? [ ] (Interviewer: if more or equal to 400 Naira go to Q3, but if less than 400 Naira, go to Q4)

3. What if the premium is 490 Naira, will you be willing to pay? [ ] 1 = yes 0 = No (Interviewer: no matter the answer, go to Q7).

4. What if the premium is 390 Naira, will you be willing to pay? [ ] 1 = yes (Q7) 0 = No (Q5)

5. What really is the maximum amount you are willing to pay for the community-based health insurance premium? [ ] (Interviewer: If more or equal to 400 Naira go to Q7, but if less than 400 Naira go to Q6)

6. The amount that you have quoted is too low, and cannot cover the cost of the premium, and so you will have to increase the amount if you really want to join the community-based health insurance scheme. So what is the final maximum amount you are willing to pay per month to join the health insurance scheme? [ ] (Interviewer: No matter the answer, go to Q7)

7. If due to inflation or other uncertainties, the premium for the community-based insurance scheme increases, what is the maximum amount you are very certain to pay bearing in mind your average monthly household income and money you spend on various items? [ ]

**58. WTP FOR OTHER HOUSEHOLD MEMBERS**

1. The price of a monthly insurance premium is 500; are you willing to pay this amount of money per household member? [ ] 1 = Yes (Q7) 0 = No (Q2) Do not know (Q2)

2. What is the maximum amount you are willing to pay? [ ] (Interviewer: if more or equal to 400 Naira go to Q3, but if less than 400 Naira, go to Q4)

3. What if the premium is 490 Naira, will you be willing to pay? [ ] 1 = yes 0 = No (Interviewer: no matter the answer, go to Q7).

4. What if the premium is 390 Naira, will you be willing to pay? [ ] 1 = yes (Q7) 0 = No (Q5)

5. What really is the maximum amount you are willing to pay for the community-based health insurance premium? [ ] (Interviewer: If more or equal to 400 Naira go to Q7, but if less than 400 Naira go to Q6)

6. The amount that you have quoted is too low, and cannot cover the cost of the premium, and so you will have to increase the amount if you really want your other household members to enrol in the community-based health insurance scheme. So what is the final maximum amount you are willing to pay per month per person for other household members to join the community-based health insurance scheme? [ ] (Interviewer: No matter the answer, go to Q7)

7. If due to inflation or other uncertainties, the premium for the community-based health insurance scheme increases, what is the maximum amount you are very certain to pay per household member bearing in mind your average monthly household income and money you spend on various items? [ ]

Would it be useful to have some sort of estimate of the possible cost of coverage for a variety of CBHI benefit packages and present these to interviewees to determine the amount that they would be willing to pay?

**59. ALTRUISTIC WTP**

*As you may know, there are some people who are too poor to pay any premium for private health insurance, but that really need to be enrolled in the scheme so that their health status would be improved, enhance their productivity and potentially decrease their poverty level.*

59a. Are you willing to contribute 500 Naira per year so that some of the poorest people could be enrolled in the private health insurance scheme per year [ ] 1 = yes 0 = no (no matter the answer, go to 59b)

59b.What is the maximum amount of money that you are willing to contribute yearly so that some of the poorest people could be enrolled in the private health insurance scheme? [ ] Naira

**SECTION 4A: PERCEIVED IMPORTANCE OF CBHI**

60. Do you believe that access to CBHI will improve your access to healthcare services in your community? 1 = yes 0= no [ ]

61. Do you believe that being enrolled in the CBHI plan will make health care more affordable for you and the members of your household? 1 = yes 0 = no [ ]

**SECTION 5: HOUSEHOLD CONSUMPTION PATTERENS**

*This section is designed to find out information to determine your socio-economic status and your households’ level of non-healthcare consumption*

62. How much did your household spend to purchase food from the market in the past one week on the various items that I will read out?

| **Item** | **Quantity** | **Who purchased** | **Amount** |
| --- | --- | --- | --- |
| **Garri** |  |  |  |
| **Beans** |  |  |  |
| **Cassava (akpu)** |  |  |  |
| **Rice** |  |  |  |
| **Fish** |  |  |  |
| **Meat** |  |  |  |
| **Others (specify)** |  |  |  |
| **Total** |  |  |  |

63. If the food items that your household produced, but also consumed in the past one week were bought from the market, how much will they cost?

| **Item** | **Quantity** | **Who purchased** | **Amount** |
| --- | --- | --- | --- |
| **Garri** |  |  |  |
| **Beans** |  |  |  |
| **Cassava (akpu)** |  |  |  |
| **Rice** |  |  |  |
| **Fish** |  |  |  |
| **Meat** |  |  |  |
| **Others (specify)** |  |  |  |
| **Total** |  |  |  |

64. Total food cost (*Enumerator add 62+ 63)* [ ] Naira

65. How often and how much do you spend on the following items? *(Enumerator: Ask for amount spent after each item and period)*

| Items | PERIOD CODES  (1 = Weekly; 2 = Monthly;  3 = Quarterly; 4 = Bi-Annually; 5 = Annually) | AMOUNT SPENT (Naira) | ***Enumerator:* ADD UP LATER (Annual Expenditure)** |
| --- | --- | --- | --- |
| a. Clothing |  |  |  |
| b. Rent |  |  |  |
| c. Durable household goods |  |  |  |
| d. Healthcare |  |  |  |
| e. Cooking fuel |  |  |  |
| f. Educational expenses |  |  |  |
| g. Other expenses (Specify) |  |  |  |
| h. TOTAL |  |  |  |

**SECTION 6: HOUSEHOLD ASSET HOLDINGS**

66. Does household own any of the following? 1 = yes 0 = no

66a. Radio [ ]

66b. Television [ ]

66c. Air conditioner [ ]

66d. Bicycle [ ]

66e. Motorcycle [ ]

66f. Car [ ]

66g. Fridge [ ]

66h. Generator [ ]

66i. Electric fan [ ]

67. Are there comments that you wish to make about the community-based health insurance scheme? [ ] 1 = yes 0 = no

68. What are they? ________________________________________________________________________________

________________________________________________________________________________________________

________________________________________________________________________________________________

Thank you

***Enumerator:* Record the time at which the interview ended ________________________________________**
